# Using electrical impedance tomography for rapid determination of starch and soluble sugar contents in *Rosa hybrida*

**DOI:** 10.1038/s41598-021-82456-1

**Published:** 2021-02-03

**Authors:** Ji Qian, Juan Zhou, Bao Di, Yang Liu, Gang Zhang, Xin Yang

**Affiliations:** 1 College of Horticulture, Hebei Agricultural University, Lekai South Street 2596, Baoding, 071000 Hebei China; 2 College of Electrical and Mechanical Engineering, Hebei Agricultural University, Baoding, 071000 Hebei China; 3 Department of Software Engineering, Hebei Software Institute, Baoding, 071000 Hebei China

**Keywords:** Plant physiology, Plant signalling, Abiotic

## Abstract

Soluble sugars and starches are important metabolites of plant life and physiological markers of plant stress response. There is an urgent need to develop a non-destructive and rapid method for determining plant starch and soluble sugar contents. Electrical impedance tomography (EIT) technology has been used to determine the physiological state and cold resistance of select plant tissues. However, so far there have been no reports on the use of EIT for the rapid estimation of soluble sugar and starch contents. In this study, EIT was used to obtain reconstructed voltage values and estimate starch and soluble sugar contents in the stems of three *Rosa hybrida* cultivars during February to May, which were grown in the Specimen Park (38° 50′ N, 115° 26′ E) of Hebei Agricultural University, Baoding City, Hebei Province, China. Stems from two of the cultivars were used for establishing regression models for starch and soluble sugar contents as functions of reconstructed voltage values. The third cultivar was used to test the accuracy of the regression models. The quadratic regression model was best for determining soluble sugar content and the logarithmic regression model was best for determining starch content. Thus, this research provided technical support for using EIT to analyze changes in physiological parameters and to rapidly estimate physiological indexes of plants. More studies were now needed to validate the results in this paper.

## Introduction

Soluble sugar and starch, the photosynthetic products of higher plants, are the main substances produced through plant carbon metabolism via the Calvin cycle and play an important role in osmotic adjustment. To a certain extent, changes in soluble sugar content reflect the adaptability of a plant to environment change^1–4^. Wang et al. (2016) showed that during the dehardening period, as the temperature naturally changed, the soluble sugar content in rose plants decreased and the starch content increased^5^. At present, starch content and soluble sugar content are usually determined using colorimetric approaches. Colorimetric approaches are easily affected by the amount of chromogenic agents, proportion of different carbohydrates in the plant, and the experimental procedures and conditions. Although measurement conditions have been optimized^6–8^, there are still shortcomings of such methods. These shortcomings include destructive sampling, extensive use of chemicals, complicated procedures, and errors caused by human operation. Therefore, it is urgent to develop a non-destructive and rapid method for determining starch and soluble sugar contents in plants.

In 1976, Swanson from the Wisconsin Madison University in the United States, first proposed the use of electrical impedance tomography (EIT), which has since attracted extensive attention. The EIT system can quickly and efficiently detect changes in the electrical characteristics of an organism, which are associated with changes in physiological state. The voltage and current across sections of the subject are measured at the surface and the internal conductivity distribution is reconstructed. To do this, a set of EIT sensors are attached around the surface of the object of study and an electric signal with an alternating current of constant amplitude is applied to the object. An electrical instrument connected to the sensors is used to measure the boundary voltage, which is then processed and output as an intuitive two-dimensional color image^9–12^.

EIT is mainly used in animal experiments and tentatively in human clinical research related to digestive, respiratory, and craniocerebral issues^13–17^. In recent years, some researchers have used EIT technology to study soil properties, wood decay, root physiological state, and cold resistance of plants^18–22^. There are few reports on the establishment of regression models for the effect of plant physiological state on EIT parameters. EIT technology can show that the impedance changes with frequency inside the object and be used to display an image of the functional state of an organ undergoing physiological changes^23^. The rapid and non-destructive determination of plant starch and soluble sugar contents can be achieved through the following procedure: the excitation signal is loaded to the object by selecting suitable electrodes; the surface voltage is detected; data are collected and processed; the image is reconstructed using algorithms; and the corresponding two-dimensional color image is displayed on a computer.

We hypothesized that the starch and soluble sugar contents in plant cells and tissues would change during the dehardening period^24^. The resulting differences in electrical conductivity could be detected using EIT technology by measuring the boundary voltage. Hence, the concentrations of the soluble sugar and starch contents could be detected. The objective of this study was to utilize the EIT imaging technology to establish regression models for the starch and soluble sugar contents as functions of reconstructed EIT boundary voltage values using data from two floribunda rose (*Rosa hybrida*) cultivars. The accuracy of the models was tested using a third floribunda rose cultivar. This study provides a technical and theoretical basis, and a mathematical model, for the rapid and non-destructive determination of starch and soluble sugar contents in plants.

## Results

### EIT images and their reconstructed boundary voltage values

**Figure 1:**
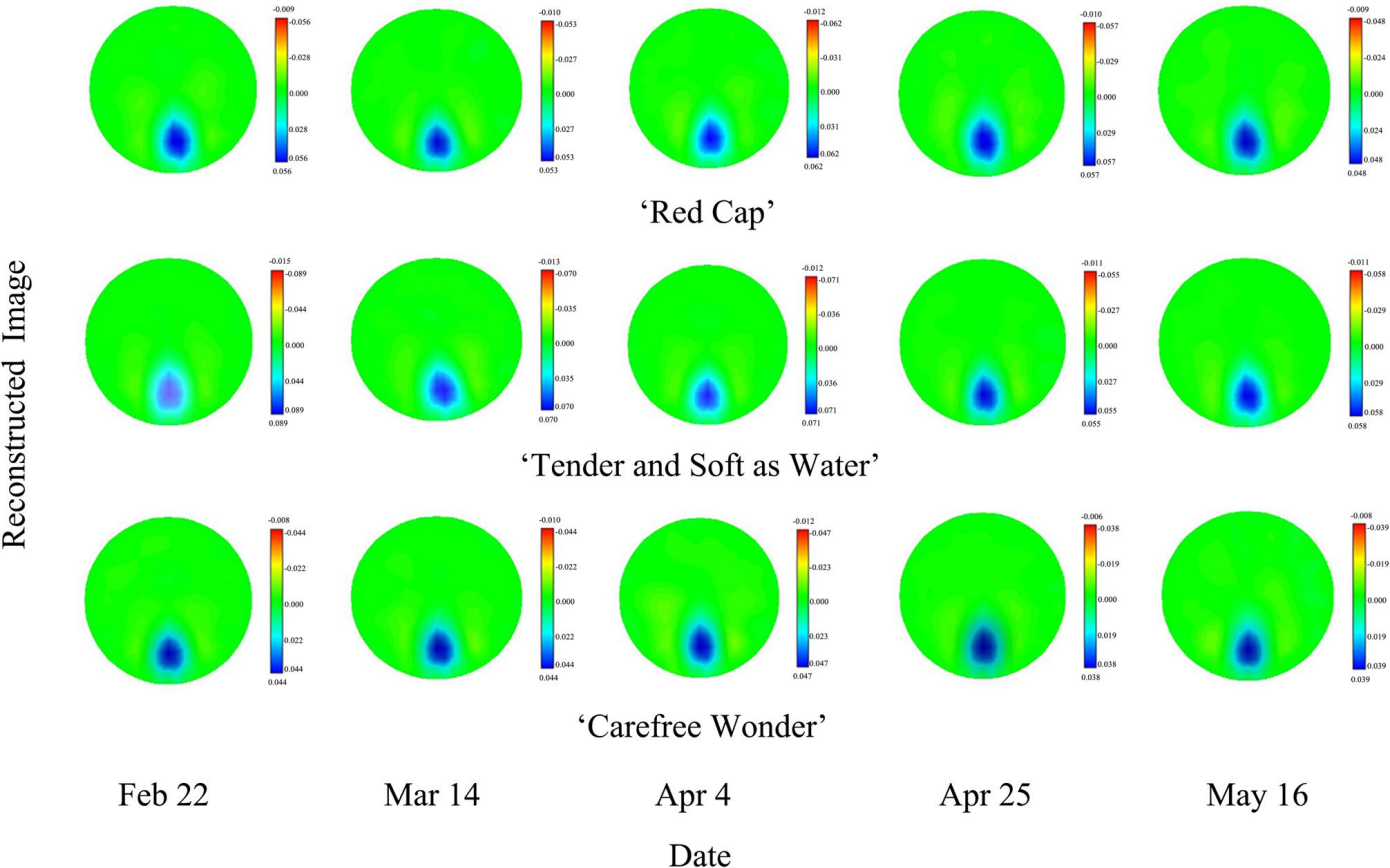
Electrical impedance tomography (EIT) images of the stems of three floribunda rose (*Rosa* hybrida) cultivars during the dehardening period.

**Figure 2:**
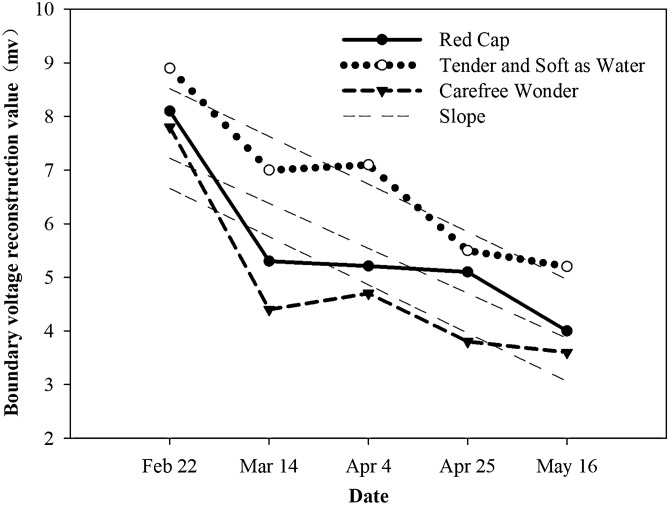
Reconstructed values of electrical impedance tomography (EIT) boundary voltages of the stems of three floribunda rose (*Rosa* hybrida) cultivars during the dehardening period.

One EIT image was selected from each of the three rose cultivars at different temperatures at each sampling time (Fig. 1). During the period of external temperature change, the EIT image accurately displayed the location, shape, and size of the rose stem as well as the distribution of resistivity. The darker the blue color, the smaller the resistivity and the corresponding reconstructed boundary voltage value. In EIT images, the pure blue color corresponds to the maximum value of the reconstructed result. Over the course of the dehardening period, the blue parts of the EIT images of the stems of the three rose cultivars gradually darkened. Figure 2 showed the change of the reconstructed EIT boundary voltage value of the stems of the three rose cultivars during the dehardening period. As shown in Fig. 2, the reconstructed boundary voltage values of the stems of the three rose cultivars showed a significant downward trend. The slopes of the curves of ‘Red Cap’, ‘Tender and Soft as Water’, and ‘Carefree Wonder’ were − 8.84, − 8.89, and − 9.20 respectively. Of these, the downward trend of ‘Carefree Wonder’ was the most significant and the reconstructed boundary voltage value of ‘Carefree Wonder’ was between 0.078 and 0.036 (Fig. 2). There were no significant differences in the reconstructed boundary voltage values between the three cultivars (*P* < 0.05).

### Changes in soluble sugar and starch contents

**Figure 3:**
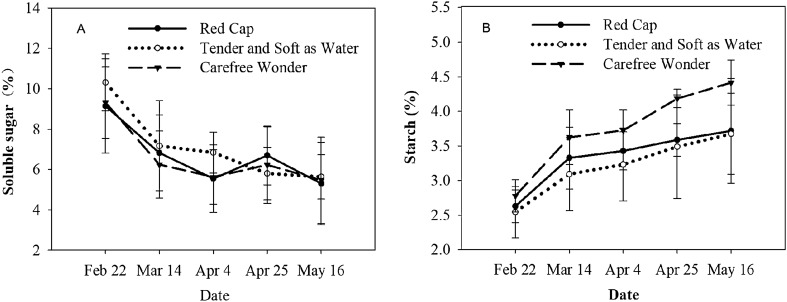
Soluble sugar content (**A**) and starch content (**B**) in the stems of three floribunda rose (*Rosa hybrida*) cultivars under natural conditions during the dehardening period.

The soluble sugar content in the stems measured with a colorimetric anthrone method. During the dehardening period, the soluble sugar content in the stems of the three rose cultivars showed an overall downward trend. The soluble sugar contents all increased slightly on April 25, and then continuously decreased, with the lowest values observed on May 16 when the last sampling was performed (Fig. 3A). The soluble sugar contents of each of the three rose cultivars were significantly lower on May 16 than at the other four sampling times (*P* < 0.01). The soluble sugar contents of ‘Red Cap’, ‘Tender and Soft as Water’, and ‘Carefree Wonder’ on May 16 were 41.99%, 45.38%, and 41.35% lower, respectively, than on February 22. However, there were no significant differences in the soluble sugar contents of the same cultivar between the March 14, April 4, and April 25 samples. Furthermore, there were no significant differences in the soluble sugar contents of the different cultivars at each sampling time.

During the dehardening period, the starch content of the stems of all three rose cultivars showed an increasing trend (Fig. 3B). The overall rate of increase in starch content of ‘Carefree Wonder’ was higher than that of the other two cultivars. The starch content of all three cultivars obviously increased during the period from February 22 to March 14, but the rate of increase was lower for the rest of the sampling period. The starch content of all three cultivars was significantly higher on May 16 than on the four other sampling dates (*P* < 0.05). The starch contents in the stems of ‘Red Cap’, ‘Tender and Soft as Water’, and ‘Carefree Wonder’ were 41.42%, 44.51%, and 58.93% higher on May 16 than on February 22. The starch content of the stem of ‘Carefree Wonder’ significantly differed between the five sampling times (*P* < 0.05). The starch contents of ‘Red Cap’ and ‘Tender and Soft as Water’ significantly differed between February 22 and the four other sampling times (*P* < 0.05); there were no significant differences in sugar content between those four sampling times. There was no significant difference in the starch content between different cultivars at each sampling time.

### Establishment of the regression models for starch and soluble sugar contents as functions of reconstructed EIT boundary voltage values

The soluble sugar and starch contents and the corresponding reconstructed EIT boundary voltage values of the two cultivars ‘Red Cap’ and ‘Carefree Wonder’ were used to establish a regression model. The measured EIT boundary voltage values ranged from 0.036 to 0.089. These values were 100 times smaller than the values of starch and soluble sugar contents. Therefore, the measured boundary voltage values were multiplied by 100. These amplified values were used together with the soluble sugar and starch contents in linear and nonlinear regression analyses. The regression model with the greatest *R*^2^ was preferentially selected as the best model. The results are shown in Table 1. The *R*^2^ values of the established linear, logarithmic, and quadratic regression models for soluble sugar content as a function of reconstructed EIT boundary voltage value, were 0.863, 0.811, and 0.897, respectively (*P* < 0.001). Thus, for soluble sugar, the quadratic regression model had the greatest *R*^2^. The *R*^2^ values of the established linear, logarithmic, and quadratic regression models for the starch content as a function of reconstructed EIT boundary voltage value, were 0.894, 0.959, and 0.935, respectively (*P* < 0.001). Thus, for starch, the logarithmic regression model had the greatest *R*^2^.

**Table 1:** Analysis of the regression for soluble sugar and starch contents as functions of reconstructed electrical impedance tomography (EIT) boundary voltage values.

Item	Index	Equation	Regression model	*R*^2^	*F*
Soluble sugar value	Boundary voltage reconstruction value	Linear	*y* = 0.868*x* + 2.125	0.863	50.527
		Logarithmic	*y* = 4.758ln(*x*) − 1.034	0.811	34.411
		Quadratic	*y* = 0.142*x*^2^ − 0.823*x* + 6.757	0.897	30.538
Starch value	Boundary voltage reconstruction value	Linear	*y* = − 0.333*x* + 5.271	0.894	67.350
		Logarithmic	*y* = − 1.917ln(*x*) + 6.634	0.959	104.964
		Quadratic	*y* = 0.059*x*^2^ − 1.032*x* + 7.189	0.935	50.132

### Testing the accuracy of the regression models for soluble sugar and starch contents as functions of reconstructed EIT boundary voltage values

The measured data from the cultivar ‘Tender and Soft as Water’ was used to test the accuracy of the regression models. The Root Mean Square Error (*RMSE*), Relative Error (*RE*), *Offset*, and the coefficient of determination (*R*′^2^) of the fitted linear regression based on measured and predicted values were compared between the established linear, logarithmic, and quadratic models. The *RE* is the percentage of the absolute error of the measurement divided by the measured value. Generally, the *RE* could well reflect the reliability of a measurement and was calculated using the Eq. (1). *RMSE* was used to estimate the deviation between the predicted value and the measured value^25^, and was usually used as a standard for assessing the predicted results of a model. The *RMSE* was calculated using Eq. (2).

1 $$ RE = \frac{{\sum\limits_{i = 1}^{n} {f(x_{i} )} - \sum\limits_{i = 1}^{n} {y_{i} } }}{{\sum\limits_{i = 1}^{n} {y_{i} } }} \times 100\% $$

2 $$ RMSE = \sqrt {\frac{1}{n}\sum\limits_{i = 1}^{n} {\left( {f(x_{i} ) - y_{i} } \right)^{2} } } $$

where *x*_i_ refers to the measured reconstructed EIT voltage values of ‘Tender and Soft as Water’; *f*(*x*_i_) was the soluble sugar content and starch content calculated by the model; *y*_i_ was the measured value of soluble sugar and starch content corresponding to *x*_i_; and *n* = 5 represents the five sampling times within the dehardening period.

The validation result of the models was shown in Table 2. The quadratic regression model for soluble sugar content prediction (*y* = 0.142*x*^2^ − 0.823*x* + 6.757) had the greatest *R*^2^ (0.897), and the smallest *RMSE* (0.811), *RE* (10.64%), and *Offset* (1.38). Therefore, it was the best model and the prediction accuracy was 89.36%. The logarithmic regression model for starch content prediction (*y* =  − 1.917ln(*x*) + 6.634) had the greatest *R*^2^ (0.959) and *R′*^2^ (0.948), and the smallest *Offset* (0.022). The *RMSE* was only 0.010 times greater than that of the linear model. Taking into consideration the *R*^2^, *RMSE*, *RE*, *Offset*, and *R′*^2^, the logarithmic model was the best model for predicting starch content, with a prediction accuracy of 93.98%.

**Table 2:** Indexes for assessing the fitting of measured and predicted values of soluble sugar and starch contents.

Item	Index	Regression model	*RMSE*	*RE* (%)	*Offset*	*R'*^2^
Soluble sugar	Boundary voltage	*y* = 0.868*x* + 2.125	1.060	11.56	3.294	0.923
		*y* = 4.758ln(*x*) − 1.034	1.197	11.26	4.293	0.876
		*y* = 0.142*x*^2^ − 0.823*x* + 6.757	0.811	10.64	1.380	0.977
Starch	Boundary voltage	*y* = − 0.333*x* + 5.271	0.202	− 5.58	0.554	0.926
		*y* = − 1.917ln(*x*) + 6.634	0.212	− 6.02	0.022	0.948
		*y* = 0.059*x*^2^ − 1.032*x* + 7.189	0.257	− 5.88	0.861	0.824

## Discussion

Carbohydrates, which exist mainly as starch and soluble sugar, were the basic substances that directly participated in plant metabolism by providing energy and carbon sources. Soluble sugars included glucose, fructose, sucrose, etc. The accumulation of starch in plants was closely related to the synthesis, transport and accumulation of soluble sugar. Thus, they were the important indicators of the intensity of life activities in plants. Many studies had shown that carbohydrates could also act as signaling molecules, connecting with hormone and nitrogen signals to form a complex signaling network. Thus, carbohydrates participated in the regulation of a series of plant metabolic activities. Carbohydrate content in plants directly affected plant growth and ecological adaptability^26–29^. In the present study, it could be seen that during the dehardening period, large amounts of soluble sugar were used for growth and the content of soluble sugar in the rose stems showed a decreasing trend. As the environmental temperature and duration of sunshine increases, more photosynthetic products were produced by new green leaves^22,30^. Meanwhile, the activities of various enzymes involved in photosynthesis were enhanced. More photosynthetic products (soluble sugars) are produced, and most of them are synthesized into starch, leading to an increase in starch content. As a result, the starch content in the stem increases, as was observed in the present study. Therefore, the content of soluble sugar and starch in the plant changed with the temperature of the external environment.

EIT technology used the electromagnetic characteristics of a tissue to reconstruct the distribution of resistivity inside the tissue and display these changed as an image. The interaction of charged ions and ionic groups allowed biological tissues to maintain a certain structure and function. Changes in physiological state result in changes to the electrical characteristics, which could be sensitively captured by the EIT system. Therefore, EIT was an imaging technology that could reflect the internal structure and function of tissues and organs. In response to changes in the external environment, plants presented a series of noticeable changes, such as in plasma membrane permeability, intracellular solutes, and cell growth and differentiation^31^. In the present study, the stem segment was exposed to the EIT saline solution for only 30 s, so the effect of the water content on the rose stem boundary voltage could be ignored.

Rose stems mainly possess fiber wood-related characteristics, which were affected by the changes in physiological parameters and in turn led to the changes in the stem dielectric properties. These dielectric changes caused differences in the reconstructed boundary voltage values. Rose stems were composed of a large number of cells of different shapes. The cells had high impedance to low frequency currents (f < 1 kHz) that only flow in the extracellular space^32^. In the present study, we used 1 kHz excitation currents. The majority of the current flows only in the extracellular spaces, and only a small part of the current passed through the cell membrane into the cell. Therefore, the extracellular impedance and part of the intracellular impedance affected the reconstructed EIT boundary voltage value.

Sugar dissolves in the cell fluid, while starch was insoluble in water. As the environmental temperature gradually increases, the carbohydrates in the stem changed accordingly. In the present study, the soluble sugar content gradually decreased while the starch content gradually increased. This affected the flow of the electric current. As a result, the stem resistivity and the reconstructed EIT boundary voltage value decreased over the course of the dehardening period. It was found that the soluble sugar and starch contents correlated well with the reconstructed EIT boundary voltage value. The *R*^2^ of the best regression models for predicting soluble sugar content and for predicting starch content were 0.897 and 0.959, respectively (*P* < 0.001). In addition, by using rose stem samples from a third cultivar for testing, both of the models were found to have decent accuracy. Therefore, it would be possible to use the reconstructed EIT boundary voltage value to predict plant soluble sugar and starch contents as a substitute for cumbersome chemical methods.

More studies were now needed to validate the results in this paper.

## Conclusions

The rapid advancement of EIT technology deems it feasible for use as a rapid and non-destructive method for the determination of physiological parameters of plants. In this study, the mathematical models for estimating soluble sugar and starch contents were preliminarily explored using the reconstructed EIT boundary voltage value of rose stems. The results provide theoretical and technical basis for the application of EIT technology in the rapid analysis of the physiological parameters of plants and their changes. This study may also promote the application of EIT technology for physiological studies of plant resistance. More studies wrer now needed to validate the results in this paper.

## Methods

### Rose plants, growth conditions, and sampling

**Figure 4:**
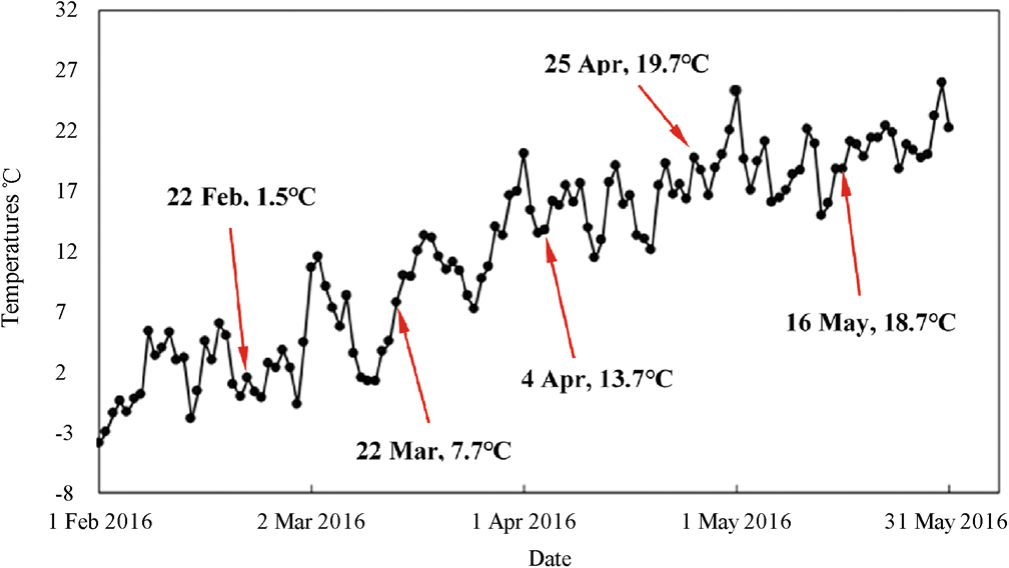
Changes in environmental temperature during the sampling period.

The biennial floribunda rose (*R. hybrida*) cultivars ‘Red Cap’, ‘Tender and Soft as Water’, and ‘Carefree Wonder’, which were grown in the Specimen Park (38° 50′ N, 115° 26′ E) of Hebei Agricultural University, Baoding City, Hebei Province, China, were used in the study. A completely randomized block design was adopted. Before the roses were planted, the soil was ploughed and barnyard manure fertilizer was applied. After planting, the soil was watered one to two times per week according to rainfall conditions. Thirty plants were grown in each plot with a spacing of 0.25 m × 0.30 m between plants. There were five replicate plots for each cultivar. A total of 450 plants were grown (30 plants/plot × 5 replicates × 3 cultivars = 450 plants). Management was applied to maintain consistent growth of the different rose cultivars. During the dehardening period from February to May 2016, the plants were sampled every 20 days and five times in total. During the sampling period, the environmental temperature was recorded by the meteorological station in the Specimen Park of Hebei Agricultural University. The daily average air temperatures of the five sampling days were 1.45 ℃, 7.7 ℃, 13.65℃, 19.65℃, and 18.75 ℃ (Fig. 4).

Five plants which grew well were selected from each cultivar. Three to four branches (1 cm diameter) with leaves, from the middle of each plant, were sampled. The samples were washed three times with tap water and a further three times with deionized water. The water on the surface of the samples was removed with absorbent papers. A part of each sample was stored in a refrigerator at 4 °C for 2 h and then used in the EIT measurement. The other part of each sample was oven dried for the conventional determination of starch and soluble sugar contents.

### Determination of reconstructed EIT boundary voltage value

In this study, we used the relatively high precision EIT system that was independently developed by researchers from the Fourth Military Medical University, Xi'an City, Shaanxi Province, China. A number of preliminary tests were conducted in order to find the excitation current that provided the best imaging and avoided noise interference. The chosen excitation current for the EIT system in this study was 1 kHz and 250 μA. A cylindrical perspex container with an inner diameter of 80 mm and a height of 80 mm was filled with 0.9% physiological saline solution (impedance does not change with frequency). Sixteen electrodes of 10 mm diameter were equidistantly installed on the wall inside the container. The model of relative excitation and adjacent measurement was adopted. The electrode plane was 25 mm from the bottom of the container and 10 mm below the surface of the physiological saline solution. Stem segments that were 70–90 mm long and 8–10 mm in diameter (excessively thin segments downgraded the image quality) with even thickness and growth status were selected. Separately, each of the five stem segments was inserted vertically in the container, 20 mm away from the wall. After the surface of the saline solution became still, 10 frames of EIT images were obtained from each stem segment. Of these, the image with the least noise interference was selected and the corresponding reconstructed boundary voltage value was used as the final datum. For the EIT, a reconstruction algorithm was used to divide the image region into several triangular elements. A total of 512 finite elements were used for image reconstruction. To improve the visual display effect, the image was smoothed. EIT imaging results are displayed using a relative gray scale: in the obtained images, a reconstruction value less than 0 indicated that there was a reduction in resistivity of the corresponding area. In the pseudo-color images, negative reconstruction values were represented by colors on the red end of the scale, and the pure red part of the image corresponded to the minimum EIT reconstruction value obtained. A reconstruction value greater than 0 indicated that there was an increase in resistivity of the corresponding region, which was represented by colors towards the blue end of the scale. The pure blue part of the image corresponded to the maximum reconstruction value. The median reconstruction value corresponded to the pure green color shown in the image. According to the algorithm, the greater the absolute value of the reconstruction results at a location, the greater the degree of variation in resistivity of the region.

**Figure 5:**
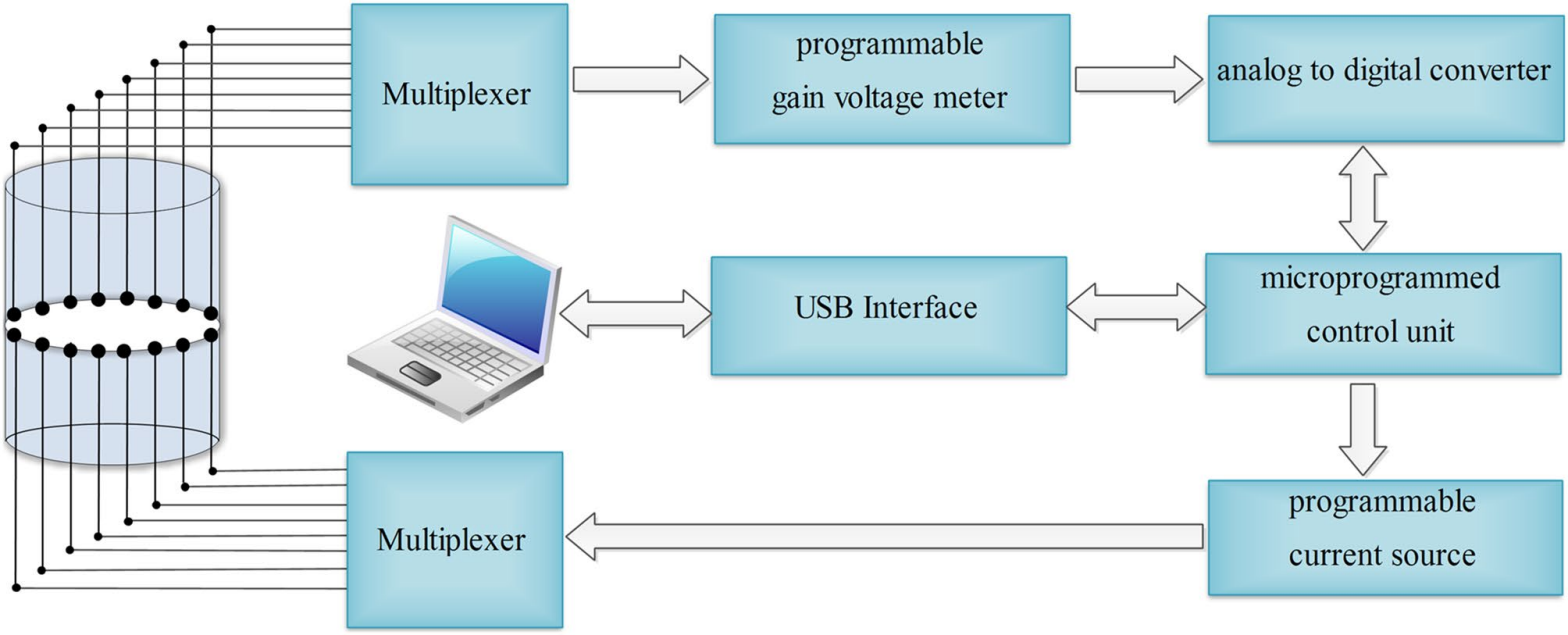
Diagram of the electrical impedance tomography (EIT) data acquisition syst.

The entire data acquisition process took only 30 s. In such a short time, the effect of the saline solution on the stem segment and the subsequent effect on the precision of the image could be ignored. In order to obtain the best quality image, we used the weighted and damped least-squares (WD-DLS) based EIT algorithm to reconstruct the image. This method was used for data processing, which eliminated the influence of background noise on the imaging quality. Meanwhile, the regularization method combined with the damped least-square reconstruction algorithm was used to deal with the ill-posed inverse problem associated with EIT. The structure of the system is illustrated in Fig. 5.

### Determination of soluble sugar and starch contents

The sampled stem segments were washed with deionized water, oven dried at 80 °C for 48 h, and then kept in desiccators before use. Soluble sugar and starch contents were determined using the anthrone colorimetric method^33^. Eight samples from each replicate were used.

To the precipitate in the tube, 2 mL water was added and the two were mixed by stirring. The tube was placed in boiling water for 15 min, and the precipitate formed a paste. After cooling on ice, 2 mL of 9.2 mol/L perchloric acid was added and the mixture was stirred well. After 15 min, 3 mL water was added, and the mixture was stirred well. The tube was then centrifuged for 10 min. The supernatant was poured into a 50 mL volumetric flask. To the precipitate in the tube, 2 mL of 4.6 mol/L perchloric acid was added and the mixture was stirred well. After 15 min, 3 mL of water was added, the mixture was stirred well, and then centrifuged for 10 min. This supernatant was also poured into the 50 mL volumetric flask. Finally, the precipitate was washed with 4 mL water and centrifuged. The centrifuged supernatant was combined with the previous supernatants collected in the 50 mL volumetric flask. Distilled water was added to make a final volume of 50 mL.

In a clean test tube, 1 mL water, and then 1 mL of extract (centrifuged supernatants collected in the 50 mL flask) were added. For the control, the test tube contained 2 mL water. Along the wall of the test tube, 5 mL 0.2% anthrone was slowly added, and the solution in the tube was mixed by shaking. The test tube was then incubated in an 80 °C water bath for 10 min to allow for color formation. After being cooled, the absorbance of the solution was measured at 620 nm. The soluble sugar and starch contents were calculated according to the following Eqs. (3) and (4):

3 $$ {\text{Soluble}}\,{\text{sugar}}\,(\% ) = \frac{C \cdot n \cdot (V/\alpha )}{{W \times 1000}} \times 100\% $$

4 $$ {\text{Starch}}\,(\% ) = \frac{C \cdot n \cdot (V/\alpha ) \times 0.9}{{W \times 1000}} \times 100\% $$

where *C* is the glucose content (μg) of the tested sample in the cuvette, which was read from a standard curve; *V* represents the total volume of extracts (mL); $\alpha$ represents the volume of extracts (mL) used in displaying color; *n* represents the fold of dilution; *W* is the dry weight of the sample (mg); and 0.9 is the glucose to starch conversion constant.

### Statistical analysis

Microsoft Excel 2016 was used to remove anomalous data according to error theory^34^. The curves of the reconstructed EIT boundary voltage values and the soluble sugar and starch contents changing with the sampling times were plotted using SigmaPlot V 10.0 (mean values were used in the figure). The correlation and variance of reconstructed EIT boundary voltage value, soluble sugar content, and starch content between the three floribunda cultivars and between the different sampling dates of each cultivar were analyzed using SPSS V 20.0 software. SPSS (Version 22.0.0.1 https://www.ibm.com/cn-zh/analytics/spss-statistics-software?lnk=hmhmmpr_bua_cnzh) was used to establish the regression models for the average soluble sugar or starch content (dependent variable *y*) of the ‘Red Cap’ and ‘Carefree Wonder’ stems as a function of their corresponding reconstructed EIT boundary voltage values (independent variable *x*). The measured data for the cultivar ‘Tender and Soft as Water’ were used in statistical analysis and verification of the accuracy of the regression models.
